# Ictal ECG-based assessment of sudden unexpected death in epilepsy

**DOI:** 10.3389/fneur.2023.1147576

**Published:** 2023-03-13

**Authors:** Adam C. Gravitis, Uilki Tufa, Katherine Zukotynski, David L. Streiner, Daniel Friedman, Juliana Laze, Yotin Chinvarun, Orrin Devinsky, Richard Wennberg, Peter L. Carlen, Berj L. Bardakjian

**Affiliations:** ^1^Institute of Biomedical Engineering, University of Toronto, Toronto, ON, Canada; ^2^Department of Radiology, McMaster University, Hamilton, ON, Canada; ^3^Department of Electrical and Computer Engineering, University of Toronto, Toronto, ON, Canada; ^4^Faculty of Health Sciences, McMaster University, Hamilton, ON, Canada; ^5^Grossman School of Medicine, New York University, New York, NY, United States; ^6^Department of Medicine, Phramongkutklao Royal Army Hospital, Bangkok, Thailand; ^7^Department of Medicine (Neurology), University of Toronto, Toronto, ON, Canada

**Keywords:** epilepsy, sudden unexpected death in epilepsy, cross-frequency coupling, ECG, signal processing, risk assessment, non-linear interaction in cardiac rhythms

## Abstract

**Introduction:**

Previous case-control studies of sudden unexpected death in epilepsy (SUDEP) patients failed to identify ECG features (peri-ictal heart rate, heart rate variability, corrected QT interval, postictal heart rate recovery, and cardiac rhythm) predictive of SUDEP risk. This implied a need to derive novel metrics to assess SUDEP risk from ECG.

**Methods:**

We applied Single Spectrum Analysis and Independent Component Analysis (SSA-ICA) to remove artifact from ECG recordings. Then cross-frequency phase-phase coupling (PPC) was applied to a 20-s mid-seizure window and a contour of −3 dB coupling strength was determined. The contour centroid polar coordinates, amplitude (alpha) and angle (theta), were calculated. Association of alpha and theta with SUDEP was assessed and a logistic classifier for alpha was constructed.

**Results:**

Alpha was higher in SUDEP patients, compared to non-SUDEP patients (*p* < 0.001). Theta showed no significant difference between patient populations. The receiver operating characteristic (ROC) of a logistic classifier for alpha resulted in an area under the ROC curve (AUC) of 94% and correctly classified two test SUDEP patients.

**Discussion:**

This study develops a novel metric *alpha*, which highlights non-linear interactions between two rhythms in the ECG, and is predictive of SUDEP risk.

## 1. Introduction

SUDEP is the sudden death of a person with epilepsy without known cause, which typically occurs after a convulsive seizure in sleep and accounts for 1 in 5 cases of epilepsy-related mortality (1–3). SUDEP often follows a generalized tonic-clonic seizure (GTCS) and results from brainstem dysfunction that impairs arousal, respiration and cardiac processes, where brainstem dysfunction may be related to suppression of activity due to spreading depolarization or other mechanisms (4–8).

Risk factors for SUDEP include increased frequency or recent history of seizure, especially tonic-clonic seizures, sub-therapeutic anti-seizure medication (ASM) levels, and lack of supervision during sleep (4). A third of epilepsy patients are not fully controlled by ASMs and many suffer ASM-related adverse effects (9). A biomarker for SUDEP could alert clinicians to recommend nocturnal monitoring or alternative treatments such as neuromodulation therapy or surgical resection (10).

Analysis of electrocardiogram (ECG) recordings may provide a biomarker of SUDEP [e.g., ventricular conduction abnormalities (11) or decreased heart rate variability (12)] but is limited by muscle-induced artifact among other issues (11–13). Single Spectrum Analysis and Independent Component Analysis (SSA-ICA) have been shown to remove artifacts from electroencephalogram (EEG) signals with simulated artifacts (14). Cross-frequency Phase-Phase Coupling (PPC) reduces sensitivity to high-amplitude noise and has been shown to be physiologically relevant for analysis of neural signals (15).

The objective of our study was to use SSA-ICA to remove artifacts from ictal ECG recordings, and assess PPC features to predict SUDEP risk. The metrics *alpha* and *theta* were derived from the PPC of a 20 s mid-seizure window.

## 2. Materials and methods

### 2.1. Study description

Our series included 9 definite-SUDEP (16) patients (sudden, unexpected death of a patient without relevant comorbidities, in which postmortem examination, including toxicology, does not reveal a cause of death other than epilepsy) and 12 non-SUDEP patients with drug-resistant focal (temporal or extratemporal lobe) epilepsy, undergoing presurgical evaluation. Patients were not on ASMs at the time of their epilepsy monitoring unit (EMU) EEG recording. The ECG recordings were acquired using the Natus/Xltek EEG system, 2-lead recordings, active electrode placed in left supraclavicular region, reference electrode on left mastoid. Apart from their definite-SUDEP designation, the following data were not available in this retrospective study: simultaneous video EEG, sleep/wakefulness states, other medications, MRI findings, or non-epilepsy medical history.

Two of the definite-SUDEP patients who succumbed to SUDEP more than 8 years after their last simultaneous EEG and ECG recordings were reserved as test cases (patients 20, 21); the remaining SUDEP patients died within 3 years of their last available recordings. Patients categorized as non-SUDEP did not die within 10 years of their last available recordings. Concurrent EEG and ECG recordings were obtained from the patients through the consortium formed by the Toronto Western Hospital, the New York University (NYU) Comprehensive Epilepsy Center, and the Phramongkutklao Royal Army Hospital (Table 1). Ictal (seizure) durations were identified from EEG scalp electrode recordings by board-certified neurologists/electroencephalographers.

**Table 1:** Table of patients.

**Patient**	**Classification**	**Age at recording**	**Sex**	**ECG sampling rate (Hz)**	**# Of GTC seizures**	**# Of non-generalized seizures**	**GTCS duration range (s)**	**Non-generalized sz duration range (s)**
1	Non-SUDEP	31	M	200	-	3	-	65–142
2	Non-SUDEP	28	M	200	-	2	-	109–232
3	Non-SUDEP	21	M	256	-	1	-	109
4	Non-SUDEP	52	F	500	-	1	-	162
5	Non-SUDEP	41	M	512	7	2	27–225	43–60
6	Non-SUDEP	35	F	512	1	-	32	-
7	Non-SUDEP	19	M	512	3	1	62–133	124
8	Non-SUDEP	62	F	512	2	6	65–84	58–79
9	Non-SUDEP	42	F	512	1	1	87	20
10	Non-SUDEP	39	F	512	-	6	-	31–115
11	Non-SUDEP	38	M	512	-	4	-	21–68
12	Non-SUDEP	28	M	512	5	3	62–110	55–75
13	SUDEP	49	M	250	-	1	-	127
14	SUDEP	30	M	250	1	-	127	-
15	SUDEP	26	F	512	-	1	-	63
16	SUDEP	13	M	256	2	1	86–186	76
17	SUDEP	21	F	256	-	1	-	241
18	SUDEP	34	M	500	-	1	-	284
19	SUDEP	43	F	512	6	2	107–394	90–129
20	SUDEP (test patient)	30	F	500	4	-	61–99	-
21	SUDEP (test patient)	47	M	200	4	3	40–53	84–371

### 2.2. Ethics approval and patient consent statement

The institutional review boards of the consortium approved the study protocol and all patients gave informed consent.

### 2.3. Data analysis

**Figure 1:**
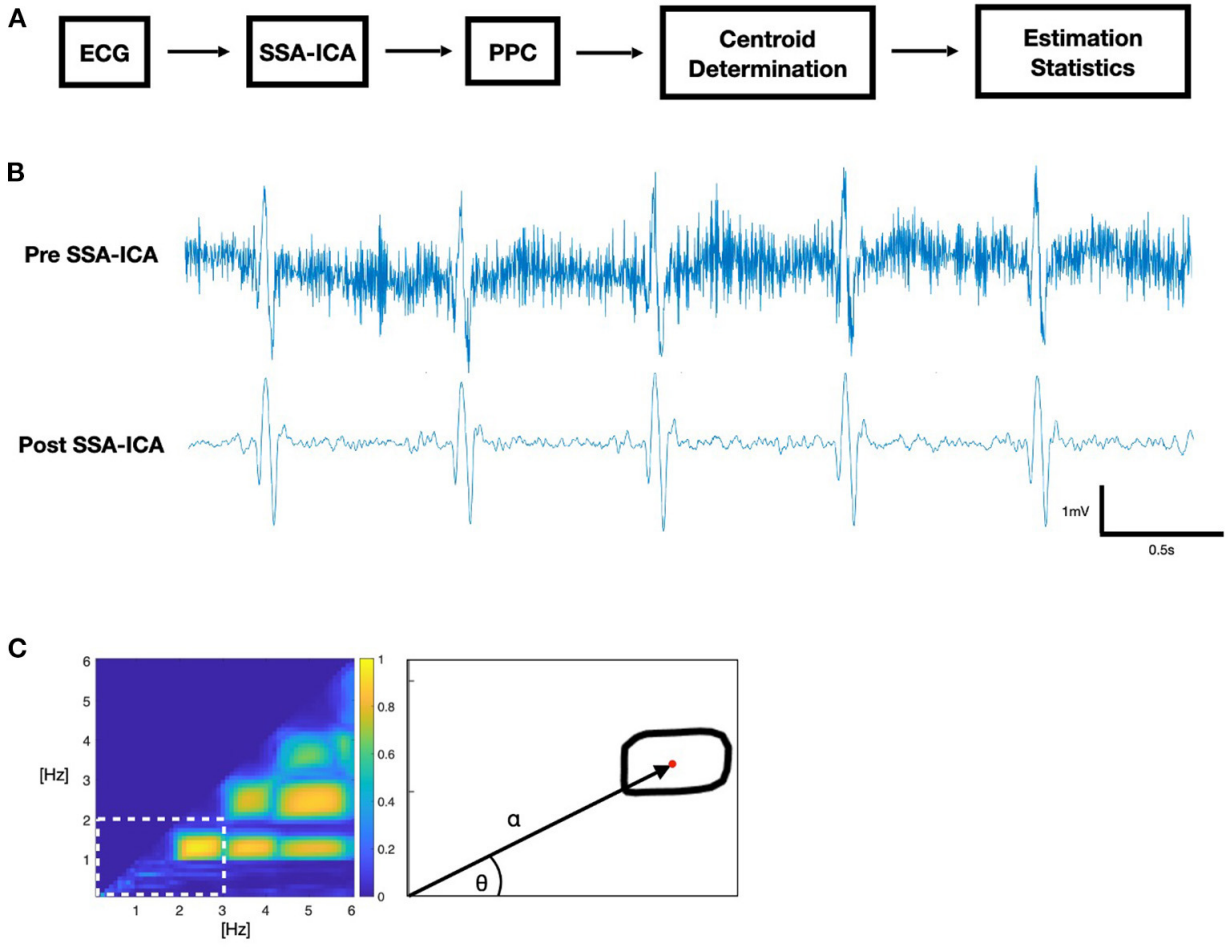
**(A)** Block diagram of ECG data analysis. **(B)** Sample ictal ECG. **(C)** Phase-phase comodulogram (PPC) feature extraction: polar coordinates *alpha* and *theta* of the contour centroid (red).

A 4-step process was used (Figure 1): (1) Applying SSA-ICA to the ictal ECG; (2) Using PPC to generate a comodulogram; (3) Analyzing the −3 dB contour of coupling power to extract the *centroid* in polar coordinates; (4) Training a logistic classifier for risk assessment. Computations were performed on the Niagara supercomputer at the SciNet HPC Consortium using Python 3.9.

#### 2.3.1. Step 1: Artifact removal

Recordings were down sampled to the lowest sample rate in the dataset, 200 Hz. We applied SSA-ICA to remove artifacts, using a 250 samples (1.25 s) window size, decomposed into 15 components. Since a residual DC-level, or drift, in the signal after the application of SSA-ICA presents as low-frequency artifact, we applied a 0.5 Hz high-pass filter to prevent ECG “baseline wander” from appearing as strongly phase-coupled artifacts in our PPC (17).

#### 2.3.2. Step 2: PPC comodulogram

A comodulogram representing the PPC of frequencies was calculated from the phase values of a continuous wavelet transformation using a morlet wavelet with center frequency of 0.8125 Hz. Phase values were compared by phase locking value (PLV) at each combination of frequencies between 0.1 and 6 Hz, using 0.1 Hz steps, where the PLV is an established measure of phase coherence (18). We used a time-averaged *n:m* PLV (19) for values of *n, m* = *1, 2, 3 … 30*, where *m* > *n*. The comodulogram indicates maximum coupling (1.0) when two frequencies within the same signal are phase-locked with respect to the quotient *m*/*n*, and no coupling (0.0) when there is no phase coherence between them. SSA-ICA was applied to the entire recording, and PPC analysis was performed for a 20 s window at the midpoint of the seizure, as identified by the electroencephalographer.

#### 2.3.3. Step 3: Contour centroid

The region of interest (ROI) for further analysis was determined by selecting the lowest-frequency contour formed by the −3 dB threshold of maximum coupling power and determining the half-power point contour using the −3 dB threshold of the *local* maximum coupling within the region where the centroid (α, θ) of this ROI is the first moment of area in polar coordinates, based on Hall (20).

#### 2.3.4. Step 4: Logistic classifier

Binary logistic regression was used to assess SUDEP risk of the seizures. Propensity scores obtained from training set alpha values were used to create a validation receiver operating characteristic (ROC) curve by varying the classification threshold from 0 to 1. An optimal propensity score threshold was selected for accuracy. Patients were then classified as SUDEP vs. non-SUDEP based on the classification of the majority of their seizures.

## 3. Results

**Figure 2:**
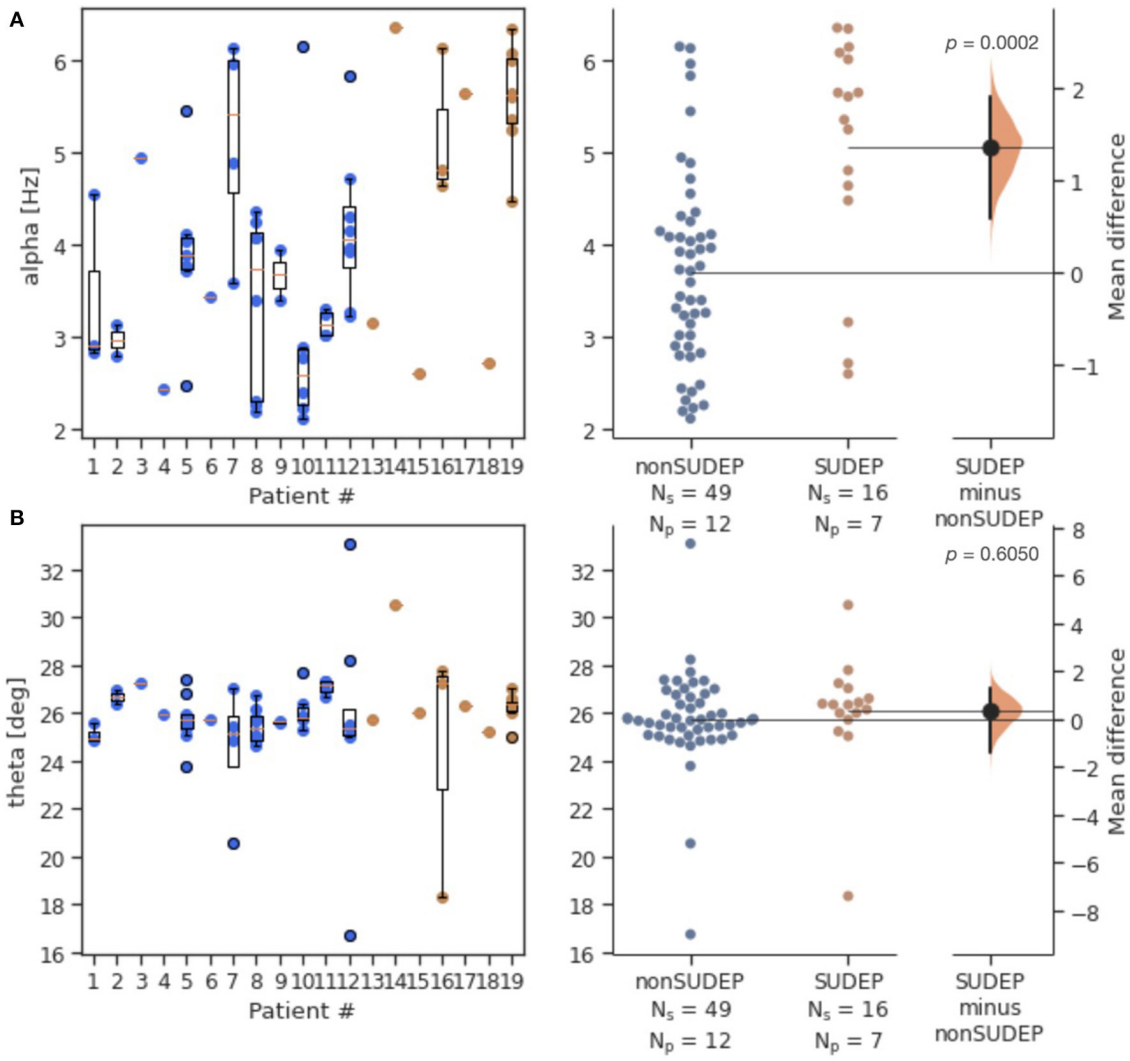
Estimation statistics of both generalized and non-generalized seizures, comparing mid-seizure 20s windows for SUDEP vs. non-SUDEP populations. Population sizes indicated by N_s_ (number of seizures), and associated N_p_ (number of patients). *Alpha* metric **(A)** and *theta* metric **(B)** of the contour centroid. Blue dots represent seizures from non-SUDEP patients. Orange dots represent seizures from SUDEP patients. Pink horizontal bars are mean values by patient.

**Figure 3:**
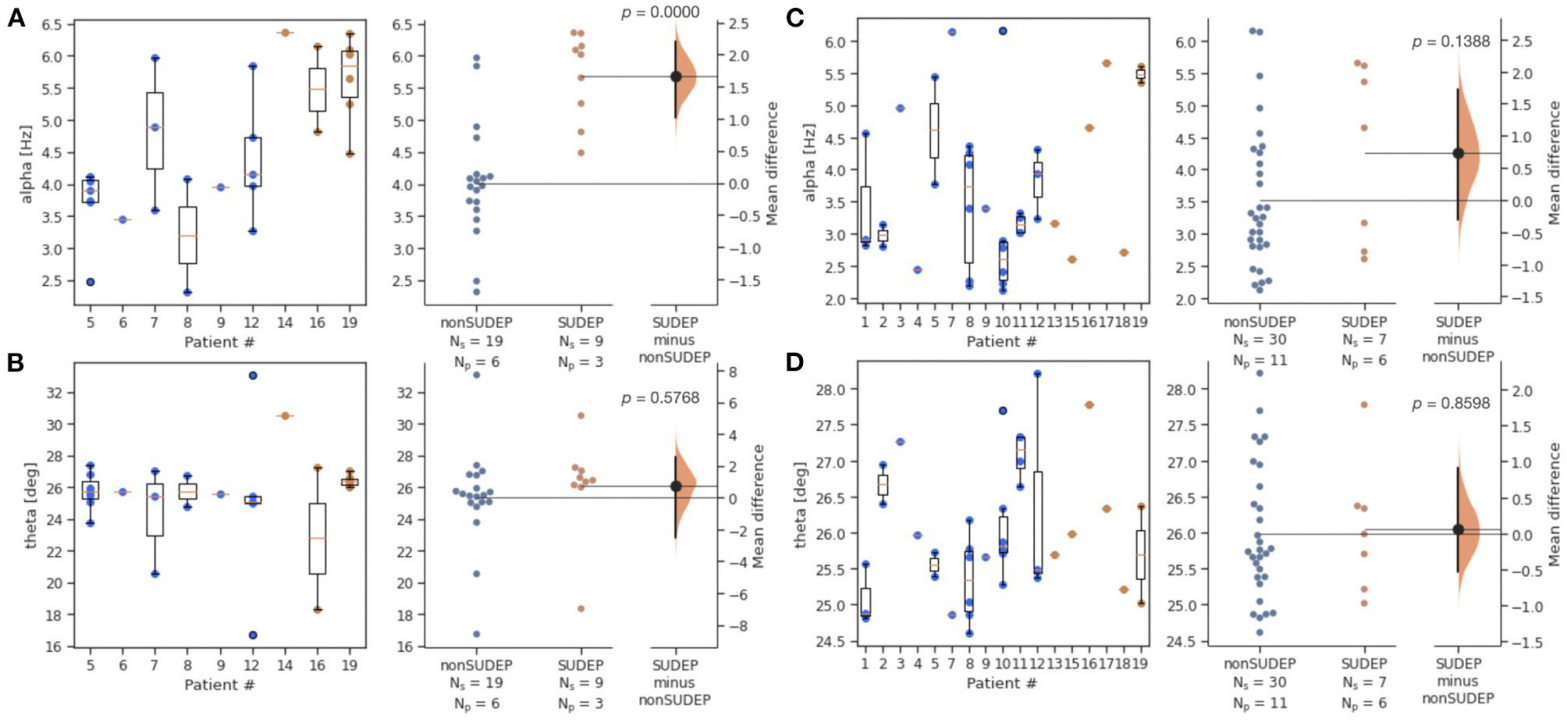
Estimation statistics for *alpha* and *theta* metrics, by two seizure types (GTCS and non-GTCS). Patient number is consistent across all figures, however not all patients have both types of seizures. N_s_ is number of seizures, N_p_ is number of patients. Blue dots represent seizures from non-SUDEP patients. Orange dots represent seizures from SUDEP patients. Pink horizontal bars are mean values by patient. **(A)** *Alpha* for GTCS only. **(B)** *Theta* for GTCS only. **(C)** *Alpha* for non-GTCS only. **(D)** *Theta* for non-GTCS only.

Alpha was significantly elevated in SUDEP compared to non-SUDEP patients (*p* < 0.0001), while there were no significant differences for theta (*p* = 0.6050). Figure 2 shows both the estimation statistics and the per-patient box plots. Estimation statistics were used to plot the overall difference between SUDEP and non-SUDEP populations. The use of estimation statistics decreases overreliance on *p-*values alone (21), and explicitly shows the effect size, range of results, and number of data points. Reported *p-*values use the Wilcoxon rank-sum test (22). Box plots show per-patient results, with one column per patient, and one data point seizure. All measures resulted in wide ranges of results on a per-patient basis, attributable to differences between seizures of a given patient. Figure 3 presents results separated by seizure type. The most significant differences are in Figure 3A, the alpha metric for GTCS (*p*<*0.0001*). The theta metric was insignificant for both types of seizures.

**Figure 4:**
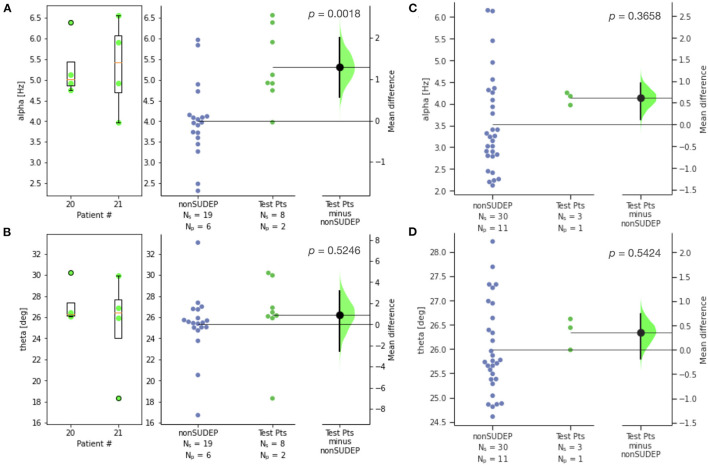
Estimation statistics for **(A)** *alpha* and **(B)** *theta* metrics for GTCS of two SUDEP patients reserved for testing, compared to those of non-SUDEP patients; **(C)** *alpha* and **(D)** *theta* metrics for non-GTCS of SUDEP patient reserved for testing (patient 21), compared to non-SUDEP patients. Patient number is consistent across all figures. N_s_ is number of seizures, N_p_ is number of patients. Blue dots represent seizures from non-SUDEP patients. Green dots represent seizures from SUDEP patients reserved for testing. Pink horizontal bars are mean values by patient.

The two SUDEP patients with recordings most distant from death were reserved for testing a classifier (patients 20, 21). Figure 4 compares *alpha* and *theta* measures for GTCS in this test set against those of non-SUDEP patients. The results are consistent with those of the training set of SUDEP GTCS seizures, where *alpha* (*p* < 0.002), but not *theta*, was significant.

**Figure 5:**
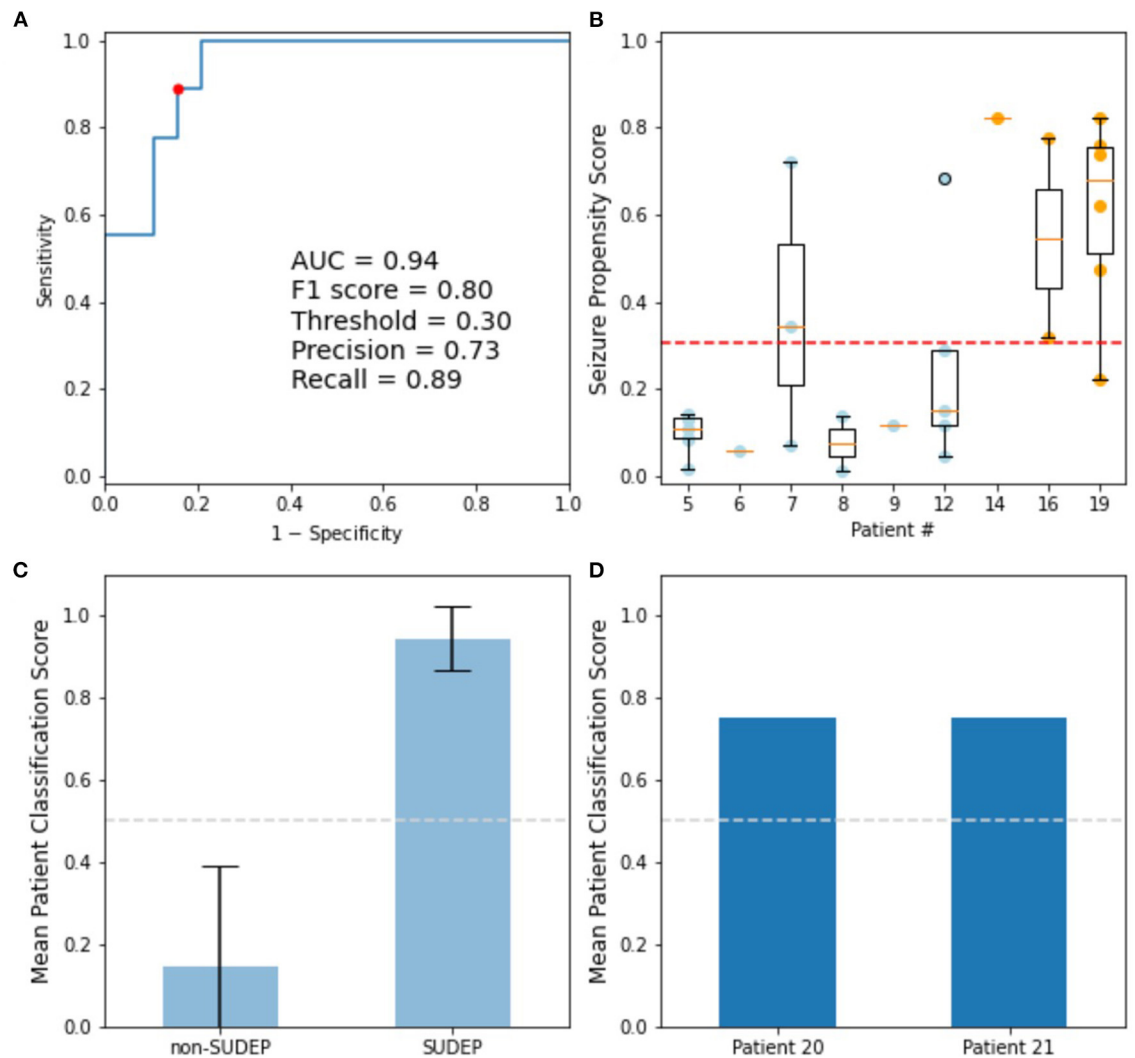
Logistic classifier trained on *alpha* determined from PPCs of GTCS. **(A)** ROC curve of training data: Accuracy optimized point (red), used to determine classifier decision threshold. **(B)** Training seizure propensity scores by patient; blue dots are non-SUDEP, orange dots are SUDEP patients. **(C)** Mean training patient classification scores, with standard deviation shown. **(D)** Mean patient classification scores for GTCS of two eventual SUDEP patients not included in training set.

For clinical application, a logistic classifier was trained on *alpha* for all the GTCS. The resulting ROC curve of training data had an area under the curve (AUC) of 94% (Figure 5). When optimizing for accuracy, the resulting F1 score is 80%, with a classifier threshold of 0.305. Each seizure was categorized as SUDEP (1.0) or non-SUDEP (0.0) relative to this threshold, and the mean patient classification scores are plotted. Error bars reflect standard deviation of each population. Both test patients are classified as SUDEP by this system.

## 4. Discussion

We describe a previously unreported PPC-based ECG metric to assess SUDEP risk using patients' GTCS features. Clinical application of this method requires only a 20-s mid-seizure ECG window for PPC analysis and *alpha* estimation. This study assesses a patient's risk of SUDEP by logistic classification using the *alpha* metrics of the majority of their seizures.

Two SUDEP test patients were withheld from classifier training. Ictal ECG recordings from the test patients 20 and 21 were acquired 10 and 8 years prior to SUDEP, respectively. Both patients resulted in mean classification scores of 0.75 (Figure 5D), thereby correctly assessed as high risk of SUDEP.

A 2010 matched-pair case-control study evaluated an extensive set of ECG features for SUDEP prediction (23). The study investigated features including peri-ictal heart rate (HR), heart rate variability (HRV), corrected QT interval (QTc), and postictal HR recovery, concluding these were not significant predictors of SUDEP. We therefore aimed to develop a novel ECG metric not derived from existing features to successfully assess SUDEP risk.

Surges et al. suggest that ECG features of SUDEP (maximal ictal HR, postictal HR recovery) identified by Nei et al. (24) can be attributed to a higher prevalence of secondarily GTCS in SUDEP patients (23, 25, 26). There were significant differences in HR and HRV between GTCS and non-GTCS, however a GTCS-only subpopulation was not investigated. A comparison between these parameters and the metrics developed in this study would be beneficial for a larger population of low-artifact ictal ECG recordings.

The assessment developed in this study controls for seizure type by considering only GTCS. The *alpha* metric in this study stands as a novel ECG feature, without derivation from previously reported ECG metrics. The *alpha* metric highlights non-linear interactions between different rhythms in the ECG. The set of ECG features used in cardiac investigations should be expanded to include this metric. Our results were obtained in ictal states only, due to limitations of the dataset in this retrospective study. Prospective studies will include sufficient pre- and post-ictal data, and explore the *alpha* metric in the context of the cardiac syncytium.

We chose a logistic regression to produce a *propensity score* (27) for each seizure from the cohorts available for this study (Figure 5B). Propensity scores can overcome an inability to pair-match in observational studies. The optimal propensity score threshold was used to categorize patients as SUDEP, or non-SUDEP, based on the classification of the majority of their seizures.

SSA-ICA was successful in decreasing artifacts throughout the recording. ECG artifacts often overlapped with frequency ranges of interest and other blind source separation methods such as ensemble empirical mode decomposition (EEMD) were less suitable under these conditions. Phase-amplitude coupling (28), where the phase of a low frequency rhythm modulates the amplitude of a higher frequency rhythm, has been used in the development of EEG-based biomarkers (29). However, PPC may be more appropriate for ECG-based biomarkers as the phases of cardiac waves are of particular interest and more robust measures than their absolute amplitudes. Measures of phase-phase coherence such as PLV are used to detect synchronization in noisy systems (19). PLV measures the cyclic relative phases between two signals. Higher artifact recordings necessitated lengthier time windows for strong coherence results. This study used a 20-s time-averaged PLV, which resulted in higher-contrast comodulograms and clearer −3 dB thresholds than were obtained for shorter durations. An artifact-tolerant measure based on PPC would enhance investigations of other cardiac-related unexplained death syndromes, such as sudden infant death syndrome (SIDS) (30), among others, and may provide an accessible measure of seizure severity.

A natural extension of this work is the application of this methodology to pre-ictal, post-ictal (29, 31) and inter-ictal ECG recordings in patients with epilepsy. Further, investigation of the PPC comodulograms could isolate the effect of waveform variability. Also, a multivariate approach incorporating EEG, respiration, and ECG would improve the risk assessment of SUDEP. A detailed analysis of the *alpha* metric would clarify its relationship with cardiac function. Analysis of a larger cohort of SUDEP patients would enhance the statistical power of these conclusions and more rapidly lead to clinical application of this work.

## Data availability statement

The data analyzed in this study is subject to the following licenses/restrictions: The anonymized datasets used in this study are available upon request. They are not publicly available due to institutional restrictions associated with the original data acquisition protocols. Requests to access these datasets should be directed to BB, berj.bardakjian@utoronto.ca.

## Ethics statement

The studies involving human participants were reviewed and approved by the Ethics Committees associated with the Toronto Western Hospital, the New York University (NYU) Comprehensive Epilepsy Center, and the Phramongkutklao Royal Army Hospital. Written informed consent to participate in this study was provided by the participants' legal guardian/next of kin.

## Author contributions

All authors listed have made a substantial, direct, and intellectual contribution to the work and approved it for publication.
