# Supplementary material for: Ictal ECG-based assessment of sudden unexpected death in epilepsy
Source: Front Neurol. 2023 Mar 13;14:1147576. doi: 10.3389/fneur.2023.1147576 (PMC10040863; doi:10.3389/fneur.2023.1147576)
Supplement: Supplementary file 1 [file Data_Sheet_1.docx]

Supplementary Material

Ictal ECG-based assessment of sudden unexpected death in epilepsy

**Adam C. Gravitis^1^, Uilki Tufa^1^, Katherine Zukotynski^2,3^, David L. Streiner^4^, Daniel Friedman^5^, Juliana Laze^5^, Yotin Chinvarun^6^, Orrin Devinsky^5^, Richard Wennberg^7^, Peter L. Carlen^1,7^, Berj L. Bardakjian^1,3*^**

^1^ Institute of Biomedical Engineering, University of Toronto, Toronto, ON, Canada

^2^ Department of Radiology, McMaster University, Hamilton, ON, Canada

^3^ Department of Electrical and Computer Engineering, University of Toronto, Toronto, ON, Canada

^4^ Faculty of Health Sciences, McMaster University, Hamilton, ON, Canada

^5^ Grossman School of Medicine, New York University, New York, NY, United States

^6^Department of Medicine, Phramongkutklao Royal Army Hospital, Bangkok, Thailand

^7^ Department of Medicine (Neurology), University of Toronto, Toronto, ON, Canada

*** Correspondence:**Berj Bardakjian
berj.bardakjian@utoronto.ca

# Phase-phase cross-frequency coupling (PPC)

The PPC comodulogram is formed by calculating the phase locking value (PLV) for each pair of frequencies from 0.1 – 6.0 Hz, in 0.1 Hz steps, after applying the high-pass filter at 1Hz. This study uses the time-averaged PLV measure^19^, with ratio integers *m*, *n* ranging 1..30:

$$PLV= \left| \frac{1}{T}\sum_{t = 1}^{T} e^{i\left( n\theta_{1}(t) - m\theta_{2}(t) \right)} \right|$$

# Contours

## Contour selection

Contours of interest were initially identified using the half-power point of the maximum value in the entire PPC. Selection criteria for the contour was that its area must be in excess of 5 pixels. The lowest-frequency contour meeting the selection criteria was selected.

Following identification of the PPC area of interest, the -3dB contour was calculated based on the maximum coupling power within that region. The threshold used to define the PLV contour is *T_r_*, calculated by taking the half power point in the traditional manner:

$$T_{r}=10\log_{10} \left( \frac{1}{2}max(PLV) \right)$$

## Contour centroid

We follow Hall et al. in the definition of a polygon centroid^20^ $\left( C_{x}, C_{y} \right)$, where:

$$C_{x}=\frac{1}{6A}\sum_{i=0}^{n-1} \left( x_{i}+x_{i+1} \right)\left( x_{i}y_{i+1}-x_{i+1}y_{i} \right)$$

$$C_{y}=\frac{1}{6A}\sum_{i=0}^{n-1} \left( y_{i}+y_{i+1} \right)\left( x_{i}y_{i+1}-x_{i+1}y_{i} \right)$$

and

$$A=\frac{1}{2}\sum_{i}^{n-1} \left( x_{i}y_{i+1}-x_{i+1}y_{i} \right)$$

**
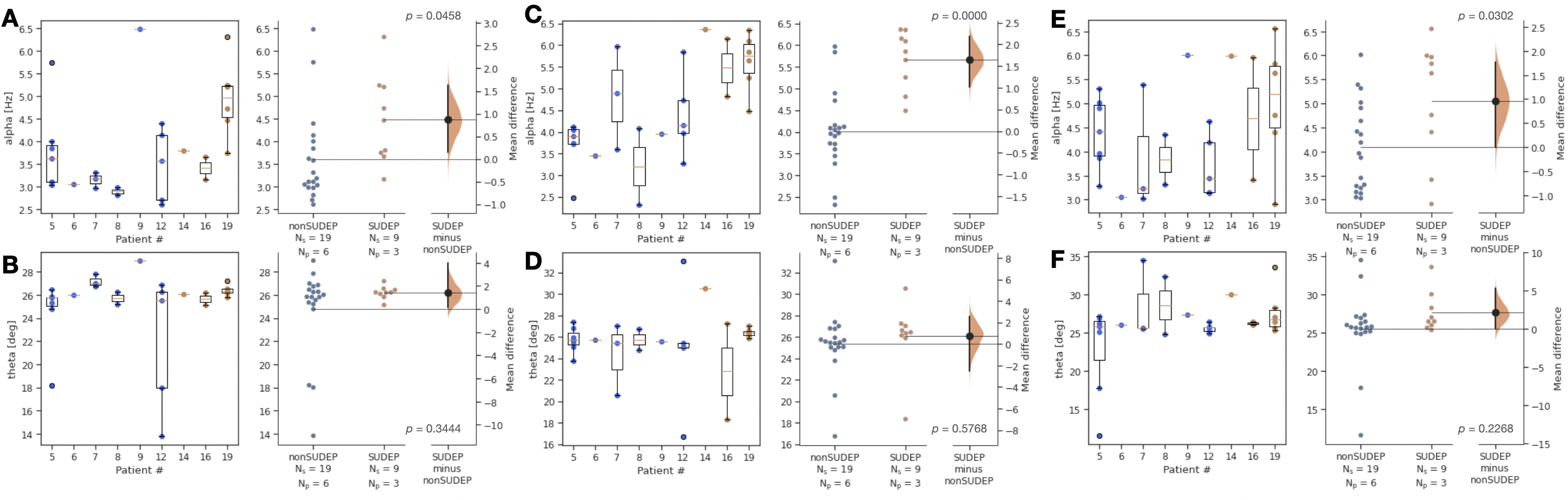
 Supplementary Figure 1:** Window selection. Comparison of *alpha* (top row) and *theta* (bottom row) for GTCS at seizure onset (**A**) and (**B**), mid-seizure (**C**) and (**D**), and termination (**E**) and (**F**). *Alpha* was most reliable with the selection of a mid-seizure window. *Theta* was insignificant for all windows.
